# Efficacy of Submandibular Gland Excision Versus Preservation During Supraomohyoid Neck Dissection for T1, T2, and N0 Oral Squamous Cell Carcinoma

**DOI:** 10.7759/cureus.74628

**Published:** 2024-11-27

**Authors:** Ranjit Karnati, Sabyasachi Parida, Saroj R Sahoo, Amaresh Mishra, Bandita Panda, Varsha Madhavnarayan Totadri, Subrat Mohanty

**Affiliations:** 1 General Surgery, Kalinga Institute of Medical Sciences, Kalinga Institute of Industrial Technology (Deemed to be University), Bhubaneswar, IND; 2 Surgical Oncology, Kalinga Institute of Medical Sciences, Kalinga Institute of Industrial Technology (Deemed to be University), Bhubaneswar, IND; 3 Research and Development, Kalinga Institute of Medical Sciences, Kalinga Institute of Industrial Technology (Deemed to be University), Bhubaneswar, IND; 4 Pediatrics Surgery, Kalinga Institute of Medical Sciences, Kalinga Institute of Industrial Technology (Deemed to be University), Bhubaneswar, IND; 5 General Surgery and Pediatric Surgery, Kalinga Institute of Medical Sciences, Kalinga Institute of Industrial Technology (Deemed to be University), Bhubaneswar, IND

**Keywords:** intraglandular lymph nodes, oral squamous cell carcinoma, submandibular gland excision, supraomohyoid neck dissection, xerostomia

## Abstract

Introduction: The present study aims to observe the efficacy of submandibular gland excision vs. preservation during supraomohyoid neck dissection (SOHND) based on the postoperative outcomes for early oral squamous cell carcinoma (OSCC) with clinically N0 neck and xerostomia.

Materials and methods: A pilot study was conducted to observe the efficacy of preservation vs. excision of the submandibular gland in early OSCC in 20 cases with an age range of 18-75 years. Intraoperative blood loss and time taken for level Ib lymph node dissection were compared. Histopathologically, lymph node yield (level Ib) was also compared. Postoperative follow-up up to four weeks and xerostomia assessment were done in both groups by using the Xerostomia Inventory score and Clinical Oral Dryness Score (CODS). Continuous and categorical data and their significance level were analyzed statistically.

Results: Routine submandibular gland excision and preservation methods were used for level Ib lymph node dissection during SOHND. Comparative assessment between the two methods revealed that surgery duration and blood loss in the test group were significantly higher (p-value < 0.001) as compared to the control group. The incidence of xerostomia in both groups did not reveal any statistical difference.

Conclusion: Submandibular gland preservation during SOHND in early OSCC leads to a slight increase in the time of dissection and blood loss is negligible and appears oncologically safer. No recurrence of intraglandular lymph nodes and zero incidence of xerostomia were the follow-up conclusions of the preservation method.

## Introduction

Oral cancer ranks sixth among the 10 most common cancer sites [1]. Oral malignancies originate in the oral tissue, including the lip, tongue, gingiva, the floor of the mouth, buccal mucosa, palate, and retromolar trigone [2]. Oral squamous cell carcinoma (OSCC) is the common histologic type (90%), followed by adenocarcinoma, whereas, verrucous carcinoma is rare [3,4]. Regular exposure to topical carcinogens (alcohol, tobacco, cigarette smoking, etc.) is considered to be a significant factor in maximum oral cancer cases [5,6]. Surgery and radiotherapy are the most common treatment modalities in OSCC cases, individually or combined. A single modality (either surgery or radiotherapy) is applicable in stage I/II cancers. In stage III, combined or multimodality treatment (surgery + radiotherapy + chemotherapy or chemotherapy + radiotherapy) is used while palliative radiotherapy and chemotherapy are used in stage IV cancers [7]. By definition, stage I oral cancer refers to a lesion less than 2 cm in size with a depth of 5 mm or less without spreading to surrounding tissue or lymph nodes. In TNM (tumor, node, and metastasis) staging, this is equivalent to T1 N0 M0. Stage 2 oral cancer refers to lesions more than 2 cm but less than 4 cm in size. In staging, it is equivalent to T2 N0 M0. In the early stages of OSCC, i.e., T1, T2, and N0, the surgical treatment of choice is wide local excision of the primary tumor with selective neck dissection, i.e., supraomohyoid neck dissection (SOHND), which involves removal of level 1, 2, and 3 cervical lymph nodes. Excision of the submandibular gland (SMG) has traditionally been an integral part of level 1B dissection as it is near lymph nodes and it was believed that sparing SMG would lead to residual unrestricted lymph nodes.

Routine excision of the SMG during level 1B dissection causes a significant decrease in saliva production, leading to xerostomia. In SMG excision, 21% of patients were reported postoperatively with xerostomia [8]. However, as the SMG does not contain intraglandular lymph nodes, removal of an uninvolved SMG may not always be necessary, particularly in cases of early-stage OSCC. This helps avoid xerostomia particularly if postoperative radiotherapy is also required [9]. The need for SMG preservation methods during level Ib lymph node dissection at SOHND arose to avoid xerostomia. It progressed with the ideology of avoiding radical treatment and balancing oncological requirements with the chance of recurrence of cancer. Thus, the present study aims to observe the efficacy of SMG excision vs. preservation during SOHND for early OSCC with clinically N0 neck, based on the postoperative outcomes, particularly, the incidence of xerostomia.

## Materials and methods

Study design and setting

A prospective, hospital-based, pilot study was conducted at the General Surgery Department, Pradyumna Bal Memorial Hospital, Kalinga Institute of Medical Sciences, Bhubaneswar, Odisha to examine the feasibility of new surgical methods in early OSCC in the period of two years (October 2020 to September 2022).

Study population

Early OSCC (T1, T2, and N0) patients and patients with clinically diagnosed node-negative status of cervical lymph nodes were the study population. Eleven patients opted for the preservation method and were in the test group, whereas nine patients underwent for routine method as a control group.

Operational definitions used in the study

SOHND is a surgical procedure to remove lymph nodes and tissue and wide local excision (WLE) is a procedure to remove a small affected area. Routine SMG excision is a procedure to remove SMG (a major salivary gland in the head and neck area). The SMG preservation method involves lymph node removal in sublevel Ib by keeping the SMG intact.

Sample size and inclusion and exclusion criteria

With patient consent, 20 consecutive OSCC cases were included in the study group. Eleven patients opted for the preservation method and were in the test group and the rest nine patients were in the control group. Patients with an age group of 18-75 years were included based on the inclusion criteria, i.e., patients presenting with early OSCC (T1, T2, and N0) and clinically diagnosed node-negative status of cervical lymph nodes. Those patients with advanced OSCC (>T2, any N, and any M), OSCC involving the floor of the mouth, patients with direct invasion of the SMG, and inoperable patients were excluded from the study.

Sampling technique

In the present study, a WLE of the primary lesion along with SOHND was done in both the test and control groups. In the test group, SMG preservation was done during the level Ib group lymph node dissection during SOHND. In the control group, routine SMG excision was done during SOHND. Intraoperatively, both groups were compared based on blood loss, and time taken for level Ib lymph node dissection. Histopathologically, lymph node yield (level Ib) was also compared between the test and control groups. Postoperative follow-up was done for up to four weeks. At four weeks, a xerostomia assessment was done in both groups using the Xerostomia Inventory score and Clinical Oral Dryness Score (CODS) [10]. Adjuvant radiotherapy was used in some patients based on lymphovascular invasion (LVI)/perineural invasion (PNI) in the histopathological examination (HPE) report after a thorough discussion in the multidisciplinary tumor board. Both the groups were again followed up at six months postoperatively, with USG of the neck to assess for any nodal recurrence and Xerostomia Inventory score and CODS for the assessment of xerostomia.

Data collection

Clinico-demographic features such as age, gender, tobacco consumption, and TNM staging were recorded. Clinical outcomes during lymph node dissection were monitored based on the dissection time, blood loss, and level of lymph node yield. Xerostomia assessment was done prospectively at four weeks and six months in both groups.

Statistical analysis

All the continuous variables were analyzed statistically and are represented in the form of mean and standard deviation. Student's t-test was used for comparative evaluation. All the categorical data are presented in frequencies and percentages. The significance level was measured by p-value < 0.05 using SPSS version 25 (IBM Corp., Armonk, NY).

Ethical consideration

Ethical approval was obtained from the Institutional Ethics Committee of Kalinga Institute of Medical Sciences, Kalinga Institute of Industrial Technology (KIIT) Deemed to be University, Bhubaneswar before patient recruitment (IEC Approval No.: KIIT/KIMS/IEC/483/2020).

## Results

**Figure 1:**
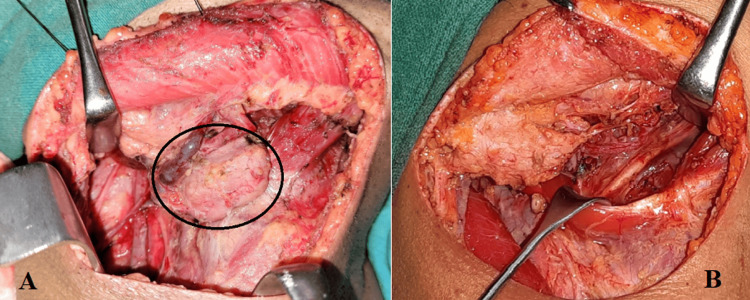
Image of supraomohyoid lymph node dissection with preservation of the submandibular salivary gland (A) vs. routine submandibular gland (SMG) excision (B) during supraomohyoid neck dissection. The circle shows the preservation of the submandibular salivary gland.

A total of 20 patients with a confirmed diagnosis of OSCC by wedge biopsy or punch biopsy as per the inclusion criteria were included during the period of two years (2020-2022). As per the American Joint Committee on Cancer 2018 staging of OSCC, eight patients belonged to T1N0 and 12 patients belonged to T2N0. Routine SMG excision during SOHND was conducted in nine patients as a control group, and 11 patients underwent SMG preservation during level Ib lymph node dissection during SOHND as a test group (Figure 1).

**Figure 2:**
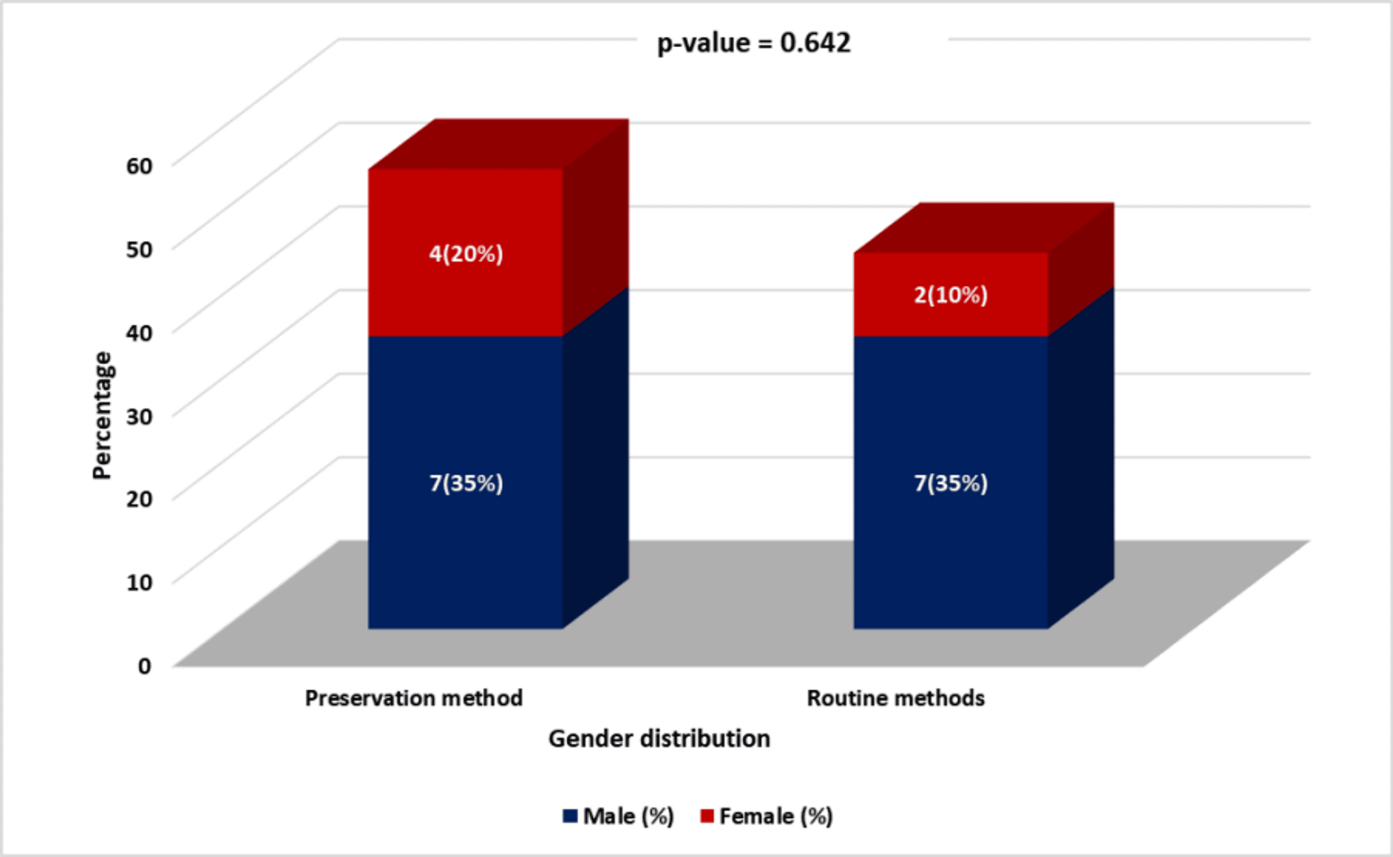
Gender distribution in the preservation method (test group) and routine method (control group). Preservation method (test group): Submandibular gland preservation during level Ib group lymph node dissection during supraomohyoid neck dissection (SOHND). Routine method (control group): Routine submandibular gland excision during SOHND.

Demographic data revealed the mean age was 52.15 ± 15.33 years with a gender ratio of 2:1 male and female, respectively. The frequency of tobacco consumption was 80% in the form of smoking or chewing (Table 1 and Figure 2).

**Table 1:** Clinical features of oral squamous cell carcinoma patients. The frequency data have been presented as numbers and percentages (in brackets).

Variables	Frequency (n = 20)
Gender: male	14 (70%)
Female	6 (30%)
Tobacco consumption	16 (80%)
TNM staging: T1N0	8 (40%)
T2N0	12 (60%)

**Figure 3:**
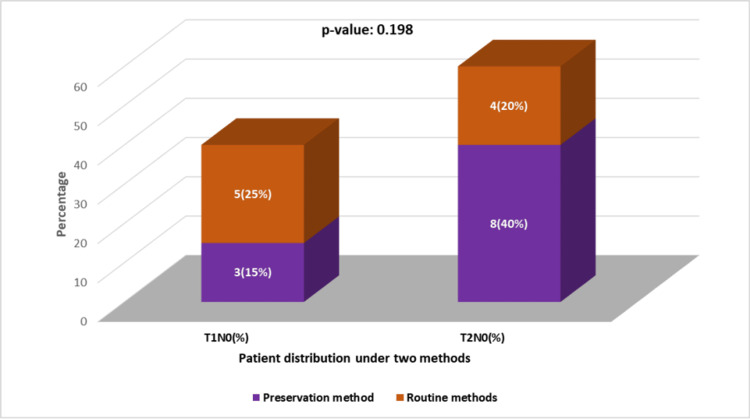
TNM (tumor, node, and metastasis) staging in the preservation method (test group) and routine method (control group).

In TNM staging, early OSCC, i.e., T1, T2, and N0, cases were included in the study. Eight cases were T1N0 and 12 cases were T2N0 (Figure 3).

Time taken for level Ib lymph node dissection in the control group ranged between 25 and 40 minutes, with a mean of 32.56 ± 5.08 minutes. In the test group, the time for level Ib lymph node dissection ranged between 45 and 65 minutes, with a mean of 50.64 ± 6.64 minutes. The test and control groups were compared based on the time taken for level Ib lymph node dissection and the result revealed that the time taken in the test group was significantly higher (p-value <0.001) as compared to the control group (Table 2).

**Table 2:** Clinical outcome during lymph node dissection. Data presented as mean and standard deviation (mean ± SD). P-value < 0.05 is significant. * Significant.

Variables	Control (n = 9)	Test (n = 11)	P-value
Time for Ib node dissection (minutes)	32.56 ± 5.08	50.64 ± 6.64	0.001*
Blood loss during Ib dissection (ml)	21.00 ± 4.74	31.64 ± 9.04	0.002*
Level Ib lymph node yield (number)	6.22 ± 2.11	7.27 ± 2.15	0.331

Blood loss during level Ib lymph node dissection ranged between 12 ml and 27 ml in the control group, with a mean of 21 ± 4.74 ml, whereas in the test group, the blood loss during level Ib lymph node dissection ranged between 18 ml and 54 ml, with a mean of 31.64 ± 9.04 ml. Blood loss in the test group was significantly higher (p-value: 0.002). The total number of lymph nodes identified in level Ib in the final histopathology report ranged between three and 12. In the control group, the level Ib lymph node yield was between three and 10, whereas in the test group, the level Ib lymph node yield was between five and 12. Lymph node yield in between the test and control group was found to be insignificant with a p-value of 0.331.

In radiotherapy, adjuvant radiotherapy was given to five out of 20 patients based on the final HPE report (PNI) and other factors after discussion in the multidisciplinary tumor board. Of the five cases, two were in the test group and three were in the control group.

Both the test and control groups were compared in the follow-up period at four-week and six-month intervals with Xerostomia Inventory scores to check for the incidence of xerostomia. The Xerostomia Inventory scale was compared between the test and control groups at four weeks of intervals and it was significant with a p-value of 0.020. Whereas, at six-month intervals, the Xerostomia Inventory scale did not show a significant comparison among the test and control groups (p-value = 0.370) (Table 3).

**Table 3:** Xerostomia assessment in post-surgery follow-ups. Data have been presented as mean and standard deviation (mean ± SD). P-value < 0.05 is significant. * Significant.

Variables	Control (n = 9)	Test (n = 11)	P-value
Xerostomia assessment at 4 weeks (Xerostomia Inventory scale)	21.11 ± 3.14	17.91 ± 3.42	0.020*
Xerostomia assessment at 6 months (Xerostomia Inventory scale)	24.89 ± 5.11	22.91 ± 3.86	0.370

No recurrence was observed in both the test and control groups at six months. But in subsequent follow-up visits, one patient (with initial osteopath report as follows: depth of invasion = 5 mm, LVI negative, PNI negative, margins free, lymph nodes not involved, T1 N0 M0) developed submental swelling at eight months postoperatively (CT of the neck showed necrotic mass lesion at the right submental region with contiguous extension to right sublingual space, inseparable from the residual tongue). However, the patient had not received adjuvant radiotherapy. Histopathology was suggestive of a recurrence of carcinoma tongue in the region of the floor of the mouth. Regional recurrence was noted for which re-surgery is followed by adjuvant radiotherapy.

Due to limited data, a significant difference could not be established in terms of xerostomia between the test and control group patients who received adjuvant radiotherapy.

## Discussion

The test group included the cases of SMG preservation during SOHND while the control group had cases with routine SMG excision. The surgery duration was higher in the preservation method than in the routine excision. This is likely because preservation of the SMG requires safeguarding of the facial artery and its branches to the submandibular gland. This dissection is tedious at times when complete lymph node dissection is intended.

Blood loss during level Ib lymph node dissection was compared between the two study groups. Blood loss was significantly higher in the test group than in the control group (p-value = 0.002). This is likely because the excision of the entire lymph node packet with an SMG and facial artery branches enables dissection in embryological planes where the chance of bleeding is much less as compared to organ-preserving dissection where lymph nodes are closely associated with the SMG.

Level Ib lymph node yield in the final histopathology report between the test and control groups was found to be insignificant (p-value = 0.331). This result is consistent with previous studies [11,12]. Ebrahim et al. did a retrospective study on SMG involvement in head and neck carcinoma and found only one sample of the gland infiltrated that too with direct extension from the primary in a total of 107 cases [11].

Another retrospective study specifically on OSCC patients showed zero involvement of SMG in a total of 115 patients. They found it as an oncologically safe procedure to save the SMG during neck dissection [13]. Javadi et al. reported one tongue squamous cell carcinoma patient having SMG out of 131 patients [14]. Another retrospective study of 69 OSCC patients showed two patients having SMG with contiguous involvement, proposing SMG preservation in early OSCC patients with preoperative node-negative disease, supporting our study [15]. At four-week and six-month intervals, using subjective questionnaires, i.e., Xerostomia Inventory scale and CODS, a comparison between the test and control groups showed a significant difference, with a slightly higher incidence of xerostomia in the control group than the test group having a p-value = 0.020. However, in the same comparison at six-month intervals, the result was insignificant (p-value = 0.370). Chen et al. assessed postoperative xerostomia between patients who underwent SMG preservation and those who underwent excision during level Ib dissection along with a control group of normal individuals of the same age and reported no difference in the scores of xerostomia between the preservation and excision groups [16,17]. This is likely because irradiation leads to a decrease or loss of SMG function. As per the USG of the neck, at four-week and six-month follow-up, no recurrence was seen in both the test and control groups. Local recurrence was observed in only one patient after eight months. This recurrence could be due to poor disease biology or a second primary lesion. As both methods were observed to be safe and recurrence was not observed in the preservation method with this low sample size, a larger multicentric study would be recommended to assess the outcomes and other benefits of SMG preservation.

Limitations

Low sample size is the major limitation of the study due to which a significant difference could not be established in terms of xerostomia between the test and control group patients who received adjuvant radiotherapy. This study was significantly affected by the COVID-19 period.

## Conclusions

Both SMG preservation and routine SMG excision during SOHND in early OSCC were performed equally. SMG preservation appeared to be safe oncologically as there were no recurrent intraglandular lymph nodes and zero incidence of xerostomia. The time of dissection was increased in the preservation method as compared to the routine method and blood loss was also more but that was negligible. To preserve or excise the SMG may be an individualized treatment choice based on patient factors and feasibility.

## References

[1] Tranby EP, Heaton LJ, Tomar SL, Kelly AL, Fager GL, Backley M, Frantsve-Hawley J (2022). Oral cancer prevalence, mortality, and costs in Medicaid and commercial insurance claims data. Cancer Epidemiol Biomarkers Prev.

[2] Dewan AK, Mitra S, Arora R, Batra U (2021). Essentials of Oral Cancer. Rajan Arora, Ullas Batra Essentials of Oral cancer.

[3] Brunicardi FC, Andersen DK, Billiar TR (2019). Schwartz's Principles of Surgery, 11th Edition. https://accessmedicine.mhmedical.com/book.aspx?bookid=2576.

[4] Schottenfeld D, Fraumeni JF (2009). Cancer Epidemiology and Prevention. Oxford University Press.

[5] Jaguar GC, Lima EN, Kowalski LP, Pellizon AC, Carvalho AL, Alves FA (2010). Impact of submandibular gland excision on salivary gland function in head and neck cancer patients. Oral Oncol.

[6] Jayant K, Deo MG (1986). Oral cancer and cultural practices in relation to betel quid and tobacco chewing and smoking. Cancer Detect Prev.

[7] Chuang SC, Jenab M, Heck JE (2012). Diet and the risk of head and neck cancer: a pooled analysis in the INHANCE consortium. Cancer Causes Control.

[8] Byeon HK, Lim YC, Koo BS, Choi EC (2009). Metastasis to the submandibular gland in oral cavity squamous cell carcinomas: pathologic analysis. Acta Otolaryngol.

[9] Jager DH, Bots CP, Forouzanfar T, Brand HS (2018). Clinical oral dryness score: evaluation of a new screening method for oral dryness. Odontology.

[10] Zhou P, Chen JX, Zhou Y, Lian CL, Yan B, Wu SG (2021). Rare metastasis to the submandibular gland in oral squamous cell carcinoma. Front Oncol.

[11] Ebrahim AK, Loock JW, Afrogheh A, Hille J (2011). Is it oncologically safe to leave the ipsilateral submandibular gland during neck dissection for head and neck squamous cell carcinoma?. J Laryngol Otol.

[12] Agarwal G, Nagpure PS, Chavan SS (2016). Questionable necessity for removing submandibular gland in neck dissection in squamous cell carcinoma of oral cavity. Indian J Otolaryngol Head Neck Surg.

[13] Javadi S, Khademi B, Mohamadianpanah M, Shishegar M, Babaei A (2021). Elective submandibular gland resection in patients with squamous cell carcinomas of the tongue. Iran J Otorhinolaryngol.

[14] Naidu TK, Naidoo SK, Ramdial PK (2012). Oral cavity squamous cell carcinoma metastasis to the submandibular gland. J Laryngol Otol.

[15] Rasool S, Manghani A, Sharma S (2024). Submandibular gland preservation in oral cavity squamous cell carcinomas: our analysis at a tertiary care hospital. Iran J Otorhinolaryngol.

[16] Chen TC, Lou PJ, Ko JY, Yang TL, Lo WC, Hu YL, Wang CP (2011). Feasibility of preservation of the submandibular gland during neck dissection in patients with early-stage oral cancer. Ann Surg Oncol.

[17] Polverini PJ, Lingen MW (2019). A history of innovations in the diagnosis and treatment of oral and head and neck cancer. J Dent Res.

